# Identification of the Immune Signatures for Ovarian Cancer Based on the Tumor Immune Microenvironment Genes

**DOI:** 10.3389/fcell.2022.772701

**Published:** 2022-03-17

**Authors:** Xiaoyan Shen, Xiao Gu, Ruiqiong Ma, Xiaoping Li, Jianliu Wang

**Affiliations:** ^1^ Department of Gynecology, Peking University People’s Hospital, Beijing, China; ^2^ Department of Oncology, Shengjing Hospital of China Medical University, Shenyang, China

**Keywords:** ovarian cancer, immune, tumor microenvironment, molecular subtype, bioinformatics analysis

## Abstract

Ovarian cancer (OV) is a deadly gynecological cancer. The tumor immune microenvironment (TIME) plays a pivotal role in OV development. However, the TIME of OV is not fully known. Therefore, we aimed to provide a comprehensive network of the TIME in OV. Gene expression data and clinical information from OV patients were obtained from the Cancer Genome Atlas Program (TCGA) database. Non-negative Matrix Factorization, NMFConsensus, and nearest template prediction algorithms were used to perform molecular clustering. The biological functions of differentially expressed genes (DEGs) were identified using Metascape, gene set enrichment analysis (GSEA), gene ontology (GO) and the Kyoto Encyclopedia of Genes and Genomes (KEGG) pathway enrichment analysis. The copy number variations (CNVs), single nucleotide polymorphisms (SNPs) and tumor mutation burden were analyzed using Gistic 2.0, R package maftools, and TCGA mutations, respectively. Estimation of STromal and Immune cells in MAlignant Tumor tissues using Expression data and CIBERSORT were utilized to elucidate the TIME. Moreover, external data from the International Cancer Genome Consortium (ICGC) and ArrayExpress databases were used to validate the signature. All 361 samples from the TCGA OV dataset were classified into Immune Class and non-Immune Class with immune signatures. By comparing the two classes, we identified 740 DEGs that accumulated in immune-related, cancer-related, inflammation-related biological functions and pathways. There were significant differences in the CNVs between the Immune and non-Immune Classes. The Immune Class was further divided into immune-activated and immune-suppressed subtypes. There was no significant difference in the top 20 genes in somatic SNPs among the three groups. In addition, the immune-activated subtype had significantly increased proportions of CD4 memory resting T cells, T cells, M1 macrophages, and M2 macrophages than the other two groups. The qRT-PCR results indicated that the mRNA expression levels of RYR2, FAT3, MDN1 and RYR1 were significantly down-regulated in OV compared with normal tissues. Moreover, the signatures of the TIME were validated using ICGC cohort and the ArrayExpress cohort. Our study clustered the OV patients into an immune-activated subtype, immune-suppressed subtype, and non-Immune Class and provided potential clues for further research on the molecular mechanisms and immunotherapy strategies of OV.

## 1 Introduction

Ovarian cancer (OV) is the third most commonly diagnosed cancer and the second leading cause of reproductive system death-related mortality in 2020 (Sung et al., 2021). Last year, 313,959 new cases of OV were reported, and 207,252 women died of this cancer worldwide. Despite the advances in surgery, chemotherapy, and target therapy for OV in recent decades, the 5-year survival rate for OV remains around 25%–35% (Lee et al., 2018). Therefore, research into potential molecular mechanisms and biomarkers to develop new anti-tumor therapy targets and to further improve the therapy response of OV patients is urgently needed.

Avoiding immune destruction is widely recognized as a key hallmark of cancer (Hanahan and Weinberg, 2011). The cancer cells can escape immune surveillance during tumor development (Chew et al., 2012; Liu and Cao, 2016) and evade immunological destruction by host immune systems (Mantovani and Sica, 2010; Hart et al., 2011; Veglia et al., 2018). Thus, many types of cancer, such as lung (Xia et al., 2021) and kidney cancer (Koh et al., 2021), are characterized by immune dysfunction. Recently, immunotherapy has emerged as a promising anti-tumor treatment strategy. Several studies have shown that immunotherapies are effective in clinical practice for cancer patients. For example, the patients with metastatic renal cell carcinoma responded to nivolumab (a programmed cell death protein 1 (PD-1) immune checkpoint inhibitor antibody) plus ipilimumab (a cytotoxic T-lymphocyte antigen-4 (CTLA-4) immune checkpoint inhibitor antibody) with acceptable toxicity (Gul et al., 2020). In patients with metastatic nonsquamous non-small cell lung cancer, pembrolizumab (an anti-PD-1 monoclonal antibody) plus pemetrexed-platinum improved overall survival (OS) and progression-free survival (PFS) with manageable safety (Gadgeel et al., 2020). In patients with advanced or metastatic esophageal squamous cell carcinoma, camrelizumab significantly improved OS, with a manageable safety (Huang et al., 2020).

Recently, evidence of a spontaneous anti-tumor immune response, satisfactory clinical response to immunotherapy, and immune evasion mechanisms have indicated that OV is an immunogenic cancer (Rodriguez-Garcia et al., 2017). Several studies have confirmed that tumor-infiltrating lymphocytes (TILs) are associated with a good prognosis of OV. Zhang *et al.* (Zhang et al., 2003) demonstrated that intratumoral T cells were observed in 54.8% of OV patients and were associated with a higher 5-year OS rate (38% vs. 4.5%). Other investigators showed that the patients with intraepithelial CD4^+^ and CD8^+^ TILs had better OS (CD4^+^ TILs, hazard ratio [HR] = 0.260; CD8^+^ TILs, HR = 0.503; both *p* < 0.05) and better PFS (CD4^+^ TILs, HR = 0.389; CD8^+^ TILs, HR = 0.478, both *p* < 0.005) than those without them (Pinto et al., 2018). Moreover, another study showed that 44.7% of OV patients had a high expression level of PD-L1 (PD-L1high), and patients with PD-L1high had a worse OS (HR = 2.877; *p =* 0.001) and PFS (HR = 1.843; *p* = 0.021) than those with low expression of PD-L1 (Zhu et al., 2017). M1 and M2 macrophages represent an important component of the tumor microenvironment (TME). M1 macrophages promoted OV metastasis through the activation of NF-κB pathway (Cho et al., 2018). Infiltration M2 macrophages was associated with poor prognosis of OV patients (Badmann et al., 2020). A high ratio of M1/M2 predicted a higher OS and PFS in OV patients (Maccio et al., 2020). Although these results offered the possibility that immunotherapy could be used for OV, the efficacy of immune checkpoint blockers in the clinic was still far from optimal. For example, the KEYNOTE-028 trial (Varga et al., 2019) showed that the objective response rate (ORR) in PD-L1-expressing advanced OV patients treated with pembrolizumab monotherapy was only 11.5%. On the other hand, another anti-PD-1 antibody, nivolumab, resulted in a 15% ORR in OV patients (Hamanishi et al., 2015). Based on the dilemma, the potential immunologic molecular mechanisms and the predictive biomarkers for immunotherapy efficacy in OV are urgently explored.

Emerging evidence suggested that the tumor immune microenvironment (TIME) changed and played a pivotal role in OV development and response to immunotherapies. For example, Huo et al. (2021) found ten TME genes related to the prognosis of OV patients. Olalekan et al. (2021) revealed the immune cell types and their roles in TME of metastatic OV by single-cell transcriptomics. Collagen type XI alpha 1 promoted OV growth and invasion by activating CAF (Wu et al., 2021). Multiple chemical agents, such as platinum derivatives, taxanes, and PARP inhibitors, regulate the interaction between tumor and stromal cells bidirectionally and extensively affect the TME (Eckert et al., 2021). Although many studies focus on the TIME of OV, the molecular mechanisms of TIME regulation of OV development remain unclear and require extensive research. In addition, a thorough understanding of the tumor immune microenvironment will guide the development of more effective immunotherapy targets for OV patients.

Therefore, we aimed to provide a comprehensive network of the immune microenvironment in OV. To achieve this goal, we divided the OV cohort of the Cancer Genome Atlas (TCGA) into Immune Class and non-Immune Class with immune signatures and analyzed the biological functions in each group. The Immune Class was further divided into immune-activated and immune-suppressed subtypes, and the gene signatures and TIME status were assessed in the three groups. Moreover, the Immune signatures of TIME were validated using the International Cancer Genome Consortium (ICGC) cohort and the ArrayExpress cohort.

## 2 Methods

### 2.1 Data Processing

Single-nucleotide polymorphisms (SNPs), clinical data, gene expression, and survival data of OV from the Cancer Genome Atlas Program (TCGA) were downloaded from the UCSC Xena platform. Gene expression data and clinical information of OV patients were obtained from the Australian Ovarian Cancer Study cohort (OV-AU). This study used TCGA data as the training data set and two OV datasets from International Cancer Genome Consortium (ICGC) and ArrayExpress as the external validation data sets.

The R package idmap1 annotated the expression data based on the Human Genome Organization’s Gene Nomenclature Committee (HGNC). We removed the mRNA expression data of the normal samples and, therefore, only expressions of tumor samples were retained to construct expression profiles for the following analyses.

We obtained the raw chip data and the corresponding annotations of the E-MTAB-62 dataset from ArrayExpress. The R packages affy (Gautier et al., 2004) and makecdfenv were used to read the raw chip data and generate the cdf package and environment. The normalized expression data using Robust Multi-array Average (RMA) method were annotated to the corresponding platform (GPL20967-3976), resulting in a gene symbol expression profile for subsequent analyses.

### 2.2 NMF Clustering

We used Non-negative Matrix Factorization (NMF) to perform molecular clustering and find the function features for each group. NMF factorizes a non-negative matrix V into two non-negative matrices, the base matrix W and the coefficient matrix H, so that V = W × H. In subsequent analyses, the coefficient matrices represent dimension-reduced matrices.

The TCGA mRNA expression profiles were subjected to NMF using the R package NMF in R environment version 4.0.5 (Gaujoux and Seoighe, 2010). The rank K of the maximum variation in cophenetic values was used as the optimal number of clustering.

### 2.3 Identification of Immune Microenvironment

We utilized the Estimation of STromal and Immune cells in MAlignant Tumor tissues using the Expression data (ESTIMATE; Becht et al., 2016) algorithm to predict the content of stromal and immune cells in each tumor sample. The stromal scores, immune scores, and ESTIMATE scores represent the ratio of stromal, immune, and the sum of both, respectively. We integrated the results of NMF and ESTIMATE to elucidate the immune microenvironments in each group.

### 2.4 Classification by NMFConsensus and NTP

We obtained and calculated the Immune Factors of the groups based on NMF and ESTIMATE. The top 150 genes of the Immune Factor were selected as Immune Genes to perform functional analysis using the Metascape database (Zhou et al., 2019). The Immune Gene expression profiles of all samples were analyzed by the NMFConsensus module of Genepattern (Reich et al., 2006) with default parameters. Samples were further divided into the immune group and non-immune group according to the result of NMFConsensus.

Nearest template prediction (NTP) is a convenient method for predicting clinical disease subtypes. To further study the immune features in OV, the GenePattern NTP module with default parameters was performed based on the activated stroma signature (Moffitt et al., 2015) of the immune group expression profile. The immune group was further divided into two subgroups: immune-suppressed and immune-activated subtypes.

### 2.5 Identification and Functional Analyses of Differentially Expressed Genes

The paired t-test of R package limma (Ritchie et al., 2015) was used to identify DEGs between the immune and non-immune groups. The absolute values of log2 [fold-change (FC)] > 1 and adjusted *p*-values < 0.01 were used to identify DEGs.

To identify the functions and relevant pathways of DEGs, we performed gene ontology (GO), and the Kyoto Encyclopedia of Genes and Genomes (KEGG) pathway enrichment analysis on DEGs using the Database for Annotation, Visualization and Integrated Discovery (DAVID) provides (Dennis et al., 2003). The GO terms of the biological process (BP) and KEGG pathways were visualized with the bubble plots. We carried out Gene Set Enrichment Analysis (GSEA) using the GSEA software to determine whether DEGs exhibit significant and consistent differences between the immune and non-immune groups (Subramanian et al., 2005).

### 2.6 Polymorphism and Tumor Mutation Burden Analyses

To analyze the copy number variation (CNV) mutations in the immune-suppressed, immune-activated and non-immune groups, we obtained CNV data for TCGA OV from GDAC Firehose. The Gistic 2.0 module from GenePattern with default parameters was conducted to identify regions with CNVs in immune-suppressed, immune-activated, and non-immune groups. The CNVs analysis results were collated and visualized using the R package maftools (Mayakonda et al., 2018). Analysis of Variance (ANOVA) tests were used to compare the CNVs among the three groups.

SNP mutation annotation format (MAF) files were downloaded from the UCSC Xena platform and processed using the R package maftools. We explored the top 20 genes of the somatic SNPs in the immune-suppressed, immune-activated, and non-immune groups by plotting the waterfall plots to identify the mutations of genes related to OV in the three groups.

We performed TMB analysis to identify the number of nonsynonymous somatic mutations in a specific genomic region in each group with TCGA OV mutation data using the R package TCGAmutations. The student’s t-test was used to compare TMB between the immune and non-immune groups.

### 2.7 TME Analysis

The analyses of the immune microenvironment are essential to identify the proportions of immune cells in tumor tissues. CIBERSORT (Newman et al., 2015) is used for the deconvolution of the expression matrix of human immune cell subtypes. This method provides gene expression signatures consisting of 22 immune cell subtypes (LM22) to estimate the abundances of immune cells. We applied CIBERSORT to analyze the immune microenvironment of expression matrices and obtained the proportion of 22 distinct immune cell types in OV tissues of different groups. We used ANOVA to compare the immune cell abundances among the three groups.

### 2.8 Sample Collection

18 OV tissues and 5 normal tissues were collected at People’s Hospital of Peking University between January 2021 and January 2022. OV tissues obtained from patients with serous adenocarcinoma. Normal ovarian tissues obtained from patients with benign gynecologic diseases who undergone bilateral salping-oophenrectomy. The protocol for this study was approved by the Ethical committee of People’s Hospital of Peking University (2021PHB239-001). Informed consent was obtained from all patients before surgery for residual tissue use.

### 2.9 Cell Lines and Cell Culture

OV cell lines SKOV3, A2780, ES2, and CAOV3 were used in the study. The human OV cells SKOV3 were incubated in McCoy’s 5A medium (Gibco, USA) contained with 10% fetal bovine serum (FBS) (Gibco, USA). The human OV cells A2780 and ES2 were incubated in RPMI 1640 medium (Gibco, USA) contained with 10% FBS. The human OV cells CAOV3 were incubated in Dulbecco’s modified Eagle medium (DMEM) medium (Gibco, USA) contained with 10% FBS. All cells were cultured at 37°C in 5% CO_2_.

### 2.10 Quantitative Real-Time PCR

Total RNA was collected with the TRIzol Reagent (Invitrogen, USA). The RNA was reversely transcribed into cDNA using a reverse transcription kit (Takara, Japan). qRT-PCR was conducted with SYBR Green PCR Master Mix (ABI, USA). GAPDH was used as a control. The expression level of genes was normalized to GAPDH. The relative expression of genes was calculated by the 2^−ΔΔCT^ method. All results are presented as the mean ± standard deviation (SD). The primer sequences used were presented in Supplementary Table S1.

## 3 Results

### 3.1 Classification of OV Based on NMF and ESTIMATE

**FIGURE 1:**
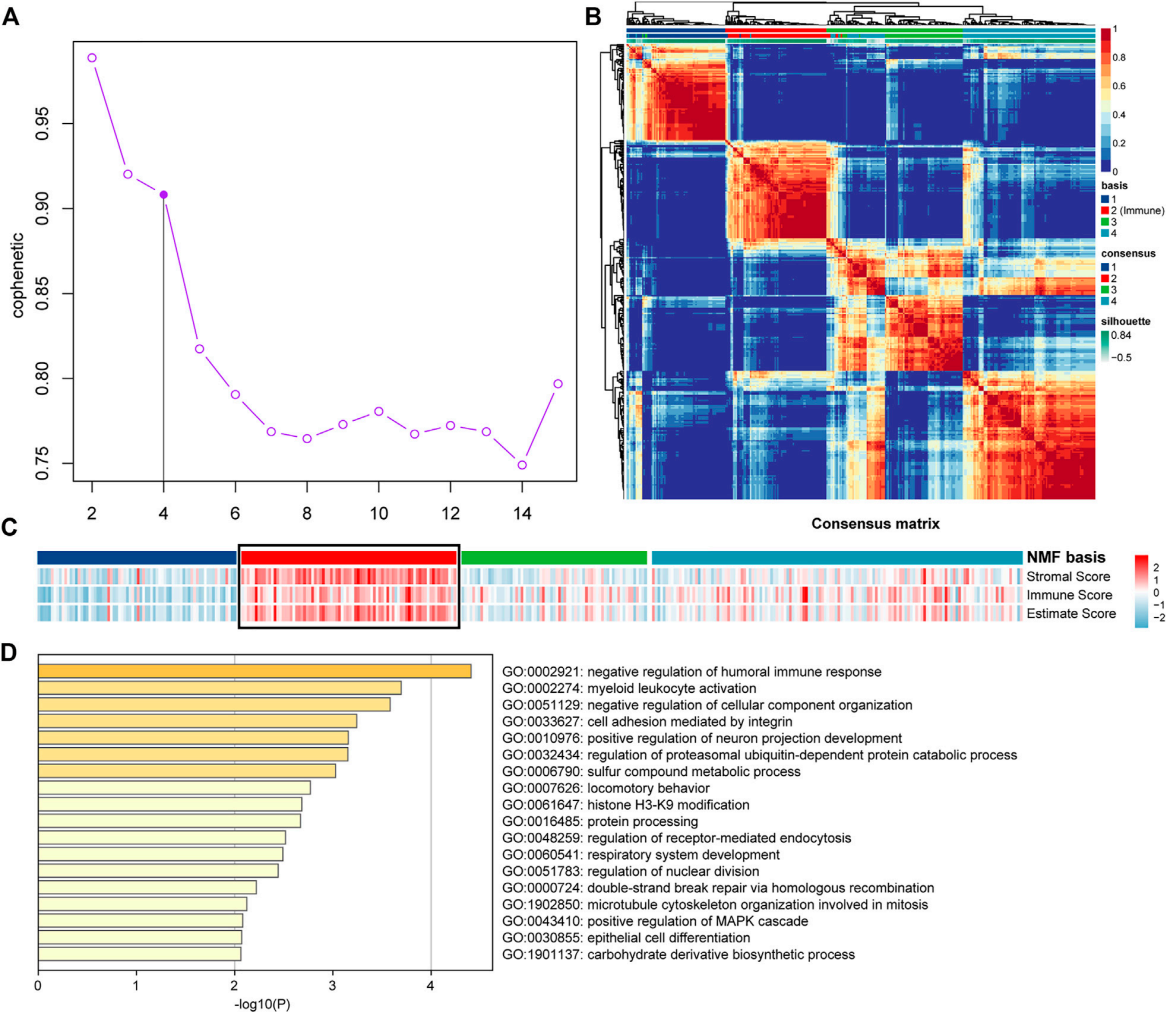
NMF and ESTIMATE analysis of OV dataset of TCGA mRNA expression profile. **(A)** The cophenetic values varied with K = 2 to 15 in NMF analysis. **(B)** The consensus map of NMF analysis results from the dimensional reduction of the original matrix and consensus analysis between modules. **(C)** The heat map of the NMF clustering results and ESTIMATE analysis results of OV. **(D)** Functional analysis of the top 150 Immune Genes of Immune Factor.

The total TCGA-OV expression profile data (361 samples) were grouped into 4 clusters by NMF. We estimate K from 2 to 15 and found that the cophenetic value changed the most when K varied from 4 to 5 (Figure 1A). Consequently, K = 4 was chosen as the optimal rank for NMF decomposition, and the decomposition results showed that the samples had stable and clear clusters (Figure 1B). We then conducted ESTIMATE analysis on all samples, and the results were integrated with the NMF results. Since cluster 2 (red module) from NMF analysis corresponded to a higher Immune Score from ESTIMATE analysis, we took cluster 2 as the Immune Factor for subsequent analysis (Figure 1C).

To ensure the accuracy of the results of Immune Factor and Immune Score from NMF analysis combined with ESTIMATE, we extracted the top 150 genes corresponding to cluster 2 from NMF analysis as Immune Genes to perform functional analysis using Metascape. The results showed that the top 150 Immune Genes from Immune Factor were significantly enriched in many immune-related biological functions, such as negative regulation of humoral immune response function and myeloid leukocyte activation function, further illustrating the accuracy of the results of Immune Factor and Immune Genes (Figure 1D).

**FIGURE 2:**
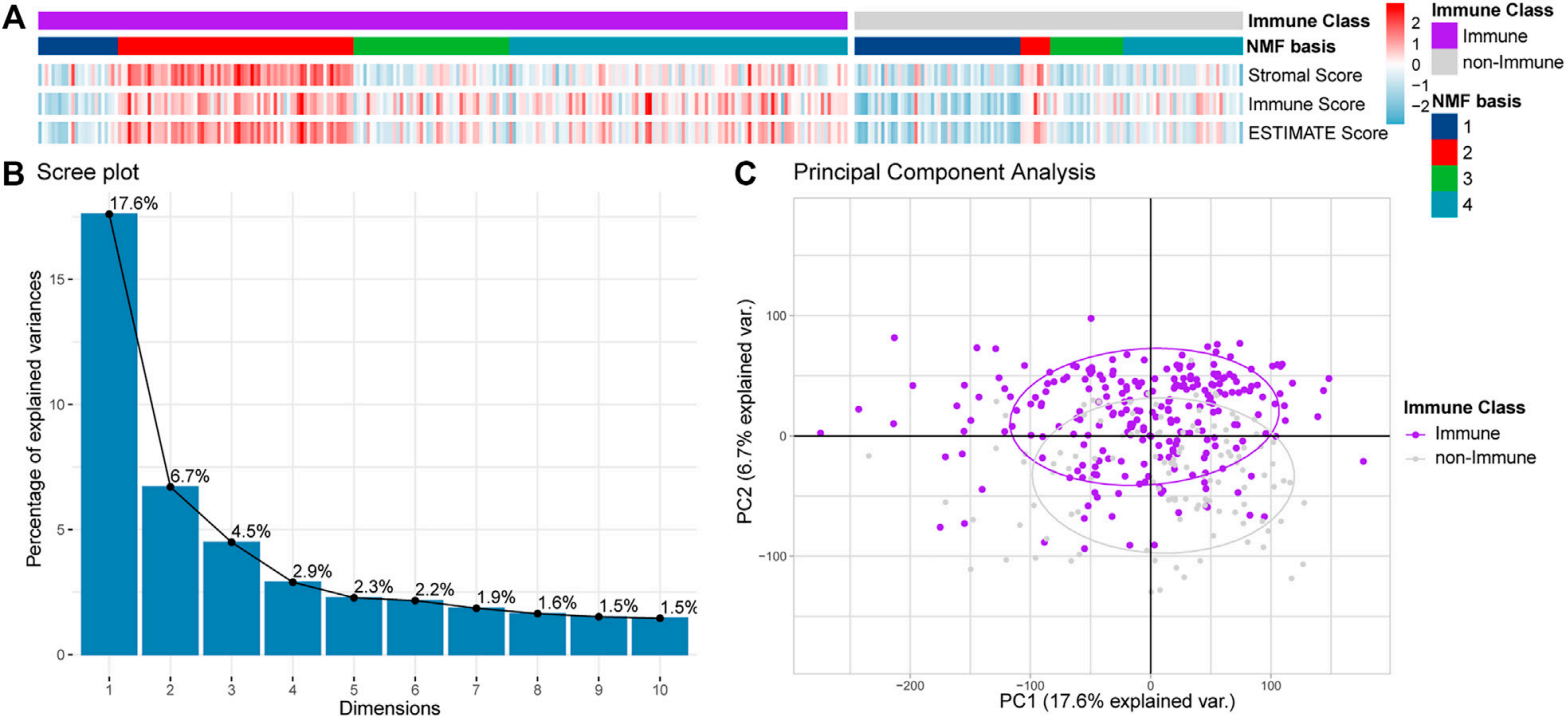
Classification of OV dataset into Immune Class and non-Immune Class. **(A)** NMFConsensus analysis with the top 150 Immune Genes. **(B,C)** The results of the Immune Class and the non-Immune Class based on principal component analysis (PCA). Each single point represents a sample.

All the samples were classified as Immune Class and non-Immune Class based on Immune Genes using NMFConsensus analysis. Figure 2A showed that the Immune Class contained almost all Immune Factors. Furthermore, the Immune Score of the Immune Class was higher than that of the non-Immune Class. In addition, principal component analysis (PCA) also showed that the Immune Class and the non-Immune Class had obvious characteristics (Figures 1B,C).

### 3.2 Functional Analyses of DEGs Between Immune Class and Non-Immune Class

**FIGURE 3:**
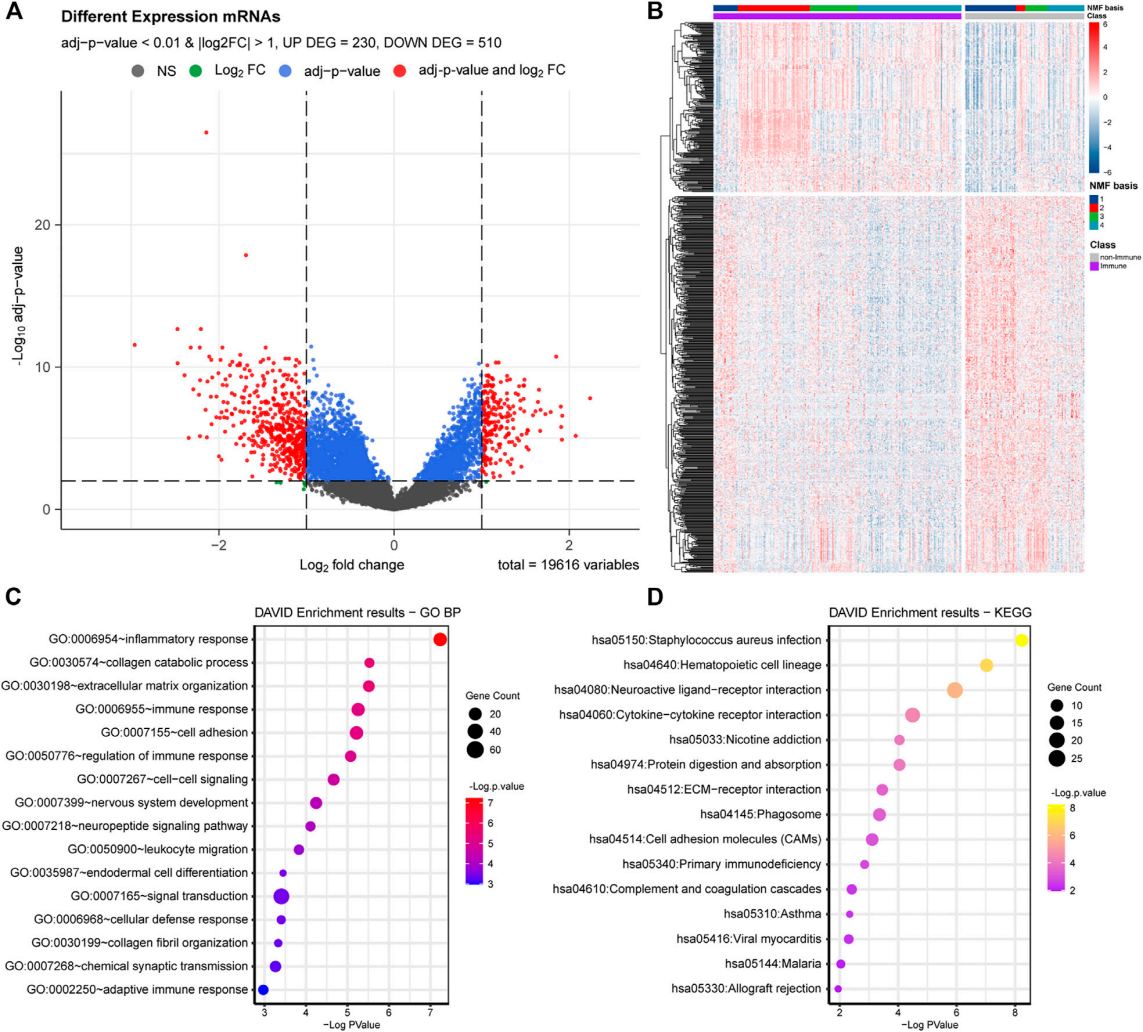
DEGs and functional analysis of Immune Class and non-Immune Class. **(A)** The distribution of DEGs is shown in the volcano plot. **(B)** NMF clustering among all the DEGs into the Immune Class and the non-Immune Class. **(C)** The top 15 enriched GO items in BP were enriched among all DEGs. **(D)** The top 15 enriched KEGG pathways among all DEGs.

We conducted functional enrichment analyses to identify the biological functions involving the Immune and non-Immune Classes. We identified 740 DEGs by comparing Immune Class with non-Immune Class (adj. *p*-value < 0.01 and |log2(FC)| > 1), including 230 upregulated and 510 downregulated DEGs (Figures 3A,B). We conducted biological functions and pathways enrichment analysis of the 740 DEGs using DAVID. The top 15 GO terms were enriched with biological processes, such as inflammatory response, immune response regulation, cell-cell signaling, leukocyte migration, signal transduction, cellular defense response, chemical synaptic transmission, and adaptive immune response, shown in Figure 3C. The top 15 entries enriched in KEGG pathways include *Staphylococcus aureus* infection, cytokine-cytokine receptor interaction, protein digestion and absorption, ECM-receptor interaction, phagosome, primary immunodeficiency, and complement and coagulation cascades (Figure 3D).

The identified DEGs, involved in many biological processes and pathways related to the immune system, suggested the reliability of the immune classification of Immune Genes by NMFConsensus. The DEGs were also involved in some interesting terms, such as extracellular matrix organization, cell adhesion, endodermal cell differentiation, signal transduction in biological processes, and neuroactive ligand-receptor interaction, nicotine addiction, protein digestion and absorption, ECM-receptor interaction, and cell adhesion molecules in KEGG pathways.

**FIGURE 4:**
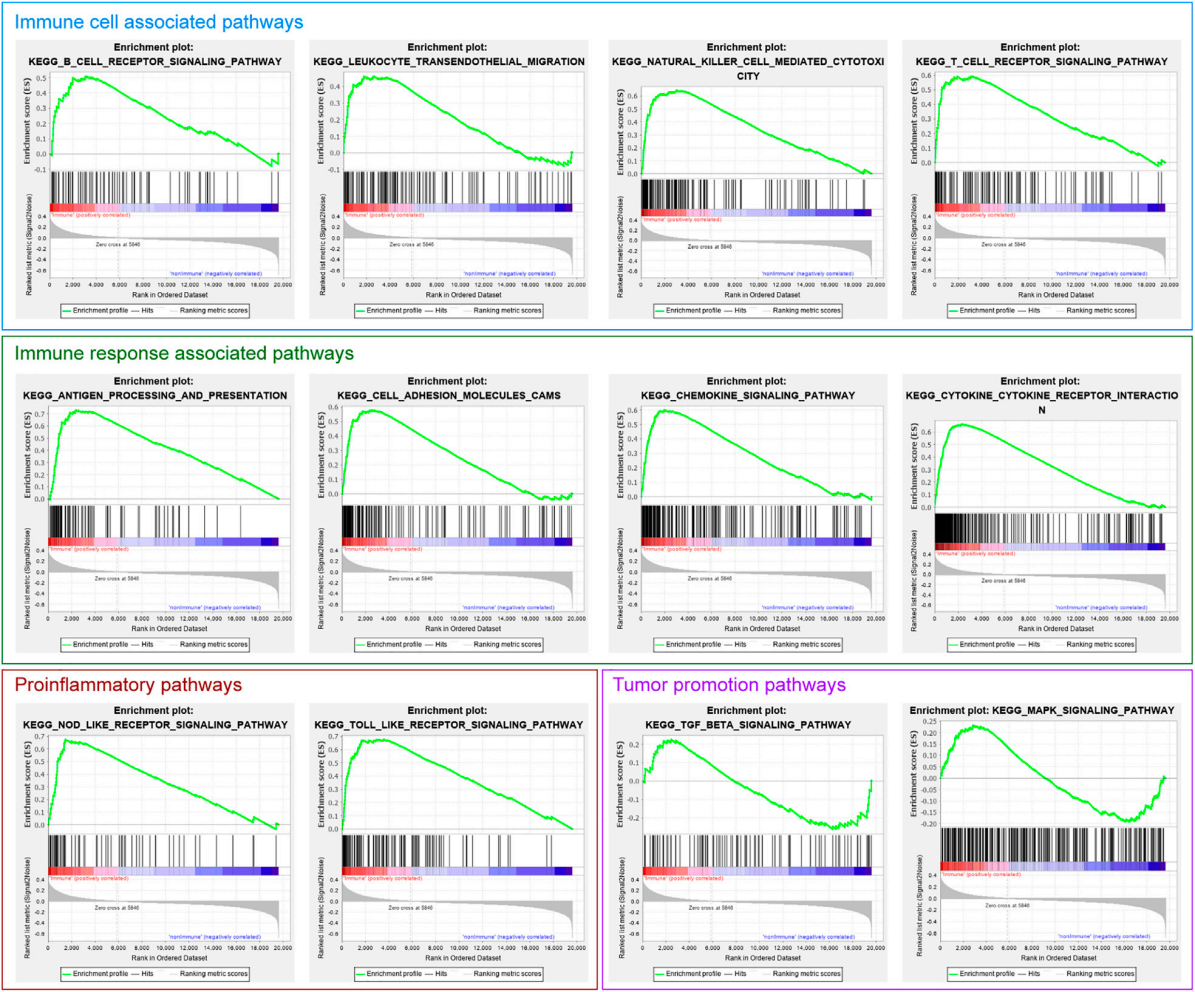
The immune-related gene set enrichment in the GSEA analysis of Immune Class and non-Immune Class.

GSEA analysis showed that numerous pathways were related to immune cell and immune response enrichment in the Immune Class, such as B cell receptor signaling pathway, leukocyte transendothelial migration pathway, natural killer cell-mediated cytotoxicity pathway, T cell receptor signaling pathway, antigen processing and presentation pathway, chemokine signaling pathway, cytokine-cytokine receptor interaction pathway (Figure 4; Table 1). Some pathways related to proinflammatory were enriched in the Immune Class, such as NOD-like receptor signaling pathway and Toll-like receptor signaling pathway. There were several pathways related to tumor promotion enriched in the Immune Class, such as MAPK signaling pathway. The results of GO and KEGG pathway analyses of DEGs identified more function and pathways related to the immune system in the Immune Class than in the non-Immune Class.

**TABLE 1:** Gene sets enriched in the Immune Class and non-Immune Class.

Gene set name	NES	NOM p-val	FDR q-val
KEGG_B_CELL_RECEPTOR_SIGNALING_PATHWAY	1.638926	0.022449	0.062037
KEGG_LEUKOCYTE_TRANSENDOTHELIAL_MIGRATION	1.578712	0.030303	0.092335
KEGG_NATURAL_KILLER_CELL_MEDIATED_CYTOTOXICITY	1.812544	0.002062	0.043273
KEGG_T_CELL_RECEPTOR_SIGNALING_PATHWAY	1.76816	0.008351	0.053921
KEGG_ANTIGEN_PROCESSING_AND_PRESENTATION	1.937901	0.001984	0.035124
KEGG_CELL_ADHESION_MOLECULES_CAMS	1.604431	0.012072	0.079889
KEGG_CHEMOKINE_SIGNALING_PATHWAY	1.753623	0.004237	0.044366
KEGG_CYTOKINE_CYTOKINE_RECEPTOR_INTERACTION	1.752546	0	0.037925
KEGG_NOD_LIKE_RECEPTOR_SIGNALING_PATHWAY	1.847274	0	0.043403
KEGG_TOLL_LIKE_RECEPTOR_SIGNALING_PATHWAY	2.030213	0	0.044734
KEGG_TGF_BETA_SIGNALING_PATHWAY	−0.87039	0.623247	0.775175
KEGG_MAPK_SIGNALING_PATHWAY	0.875658	0.668763	0.851398

NES: normalized enrichment score; NOM: nominal; FDR: false discovery rate. Gene sets with NOM p-val < 0.05 and FDR q-val < 0.25 are considered as significant.

### 3.3 TCGA OV Dataset Characteristics Analysis

**FIGURE 5:**
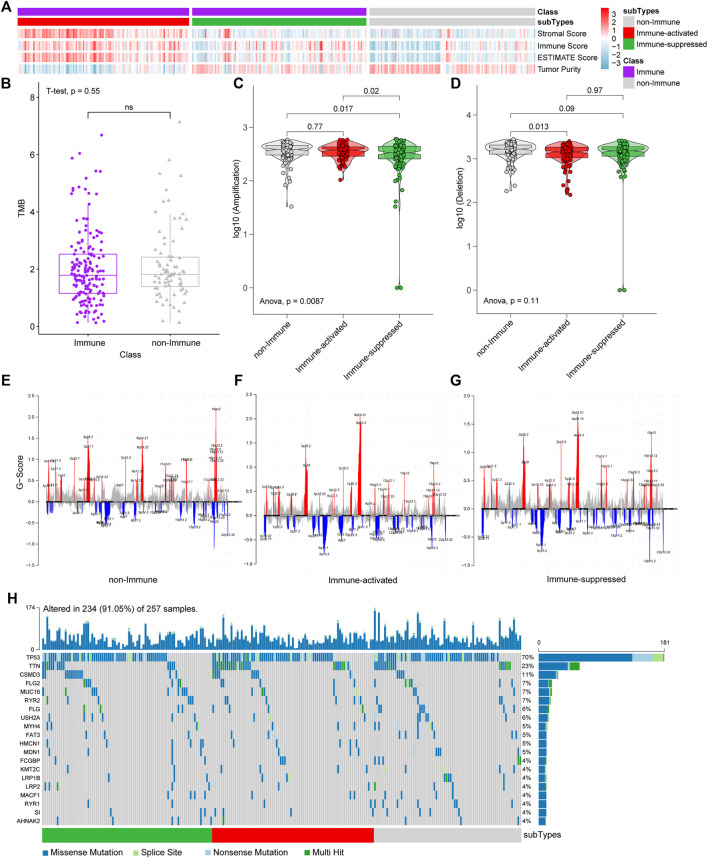
NTP, CNVs, and SNPs analyses among immune-suppressed subtype, immune-activated subtype, and non-Immune Class. **(A)** TCGA OV dataset was divided into the immune-suppressed subtype, the immune-activated subtype, and the non-Immune Class. **(B)** The TMB was estimated in the Immune Class and the non-Immune Class. **(C)** Statistical analyses of CNV amplification among the immune-suppressed subtype, the immune-activated subtype, and the non-Immune Class. **(D)** Statistical analysis of CNV deletion among the immune-suppressed subtype, the immune-activated subtype, and the non-Immune Class. **(E–G)** CNV analyses in the non-Immune Class **(E)**, the immune-activated subtype **(F)** and the immune-suppressed subtype **(G)** (red for CNV amplification and blue for CNV deletion, some significant genes were marked). **(H)** Waterfall diagram of SNP analyses.

We applied the NTP algorithm to identify immune-suppressed and immune-activated subtypes in the Immune Class identified by integrated DEGs and classification analyses. The TCGA OV dataset was divided into the non-Immune Class, immune-suppressed, immune-activated subtypes according to their immune microenvironments. The decrease in immune-related scores and the increase in Tumor Purity from the Immune activated subtype and the Immune suppressed subtype to non-Immune subtype were observed (Figure 5A), which is consistent with the features of the three subtypes.

We analyzed CNVs, SNPs, and TMB among the subgroups to identify genetic variations among subgroups. CNV amplifications differ significantly between the Immune-suppressed subtype and non-Immune Class (*p* = 0.017) and Immune-activated subtype (*p* = 0.02) (Figure 5C). A difference in CNV deletion was observed between Immune Activated subtype and non-Immune Class (*p* = 0.013) (Figure 5D). There were significant differences in the amplification and deletion of genes between the non-Immune Class and the Immune Class. The significantly amplified genomic regions in both subtypes included 8q24.21 (with the highest G score), 8q24.3, 3q26.2, 19q12, 3q29. Meanwhile, the genomic regions with a significant deletion in both subtypes included 5q12.1, 19p13.3. The amplification of 19q12, 3q27.1, and 8q24.22 was frequently associated with the non-Immune Class, and the deletion of 18q23 was more frequently occurring in this class (Figures 5E–G). However, the non-Immune Class did not differ from the Immune Class in TMB (*p* = 0.55) (Figure 5B) and not from the Immune-activated subtype in CNVs amplification (*p* = 0.77). In addition, no significant difference of top 20 genes in TCGA OV somatic SNPs among the three groups was found (Figure 5H), which agrees with the TMB results (Figure 5B).

### 3.4 Different Tumor Immune Microenvironments Among Subtypes

**FIGURE 6:**
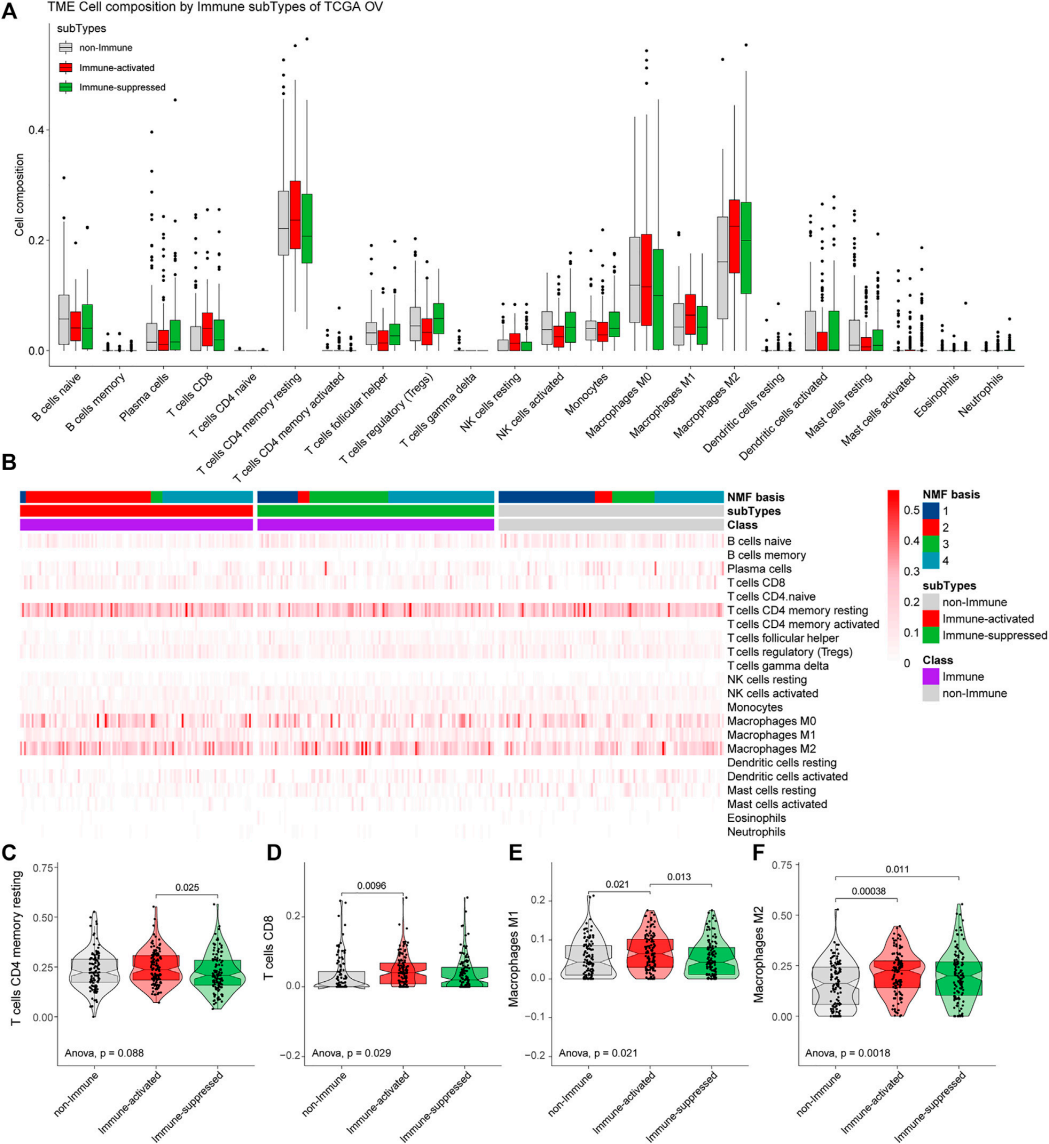
Estimation of immune microenvironments in TCGA OV samples. **(A)** The proportions of specific 22 immune cells in the non-Immune Class, the Immune-suppressed subtype, and the Immune-activated subtype. **(B)** The proportions of specific 22 immune cells in each group from NMF analysis. **(C–F)** The differential proportions of memory CD4 resting T cells **(C)**, CD8 T cells **(D)**, M1 macrophages **(E)**, and M2 macrophages **(F)** among the non-Immune Class, the Immune-suppressed subtype, and the Immune-activated subtype.

We analyzed the tumor immune microenvironments in each subgroup with CIBERSORT. We found that the most common immune cell type was CD4 memory resting T cells, followed by M2 macrophages and M0 macrophages (Figure 6A). We further estimated the proportions of the specific 22 immune cells in different groups from the NMF analysis (Figure 6B).

The proportions of the main immune cells were different among the non-Immune Class, the Immune-suppressed subtype, and the Immune-activated subtype. Compared with the Immune-suppressed subtype, we found higher proportions of CD4 memory resting T cells in the Immune-activated subtype (*p* = 0.025). Compared with the non-Immune Class, we found higher proportions of D8 T cells in the Immune-activated subtype (*p* = 0.0096). The Immune-activated subtype had a higher proportion of M1 macrophages than the Immune-suppressed subtype (*p* = 0.013) and the non-Immune Class (*p* = 0.021), and increased proportions of M2 macrophages than the Immune-suppressed subtype (*p* = 0.011) and the non-Immune Class (*p* = 0.00038) (Figures 6C–F).

### 3.5 External Validation of the Mutation Genes

**FIGURE 7:**
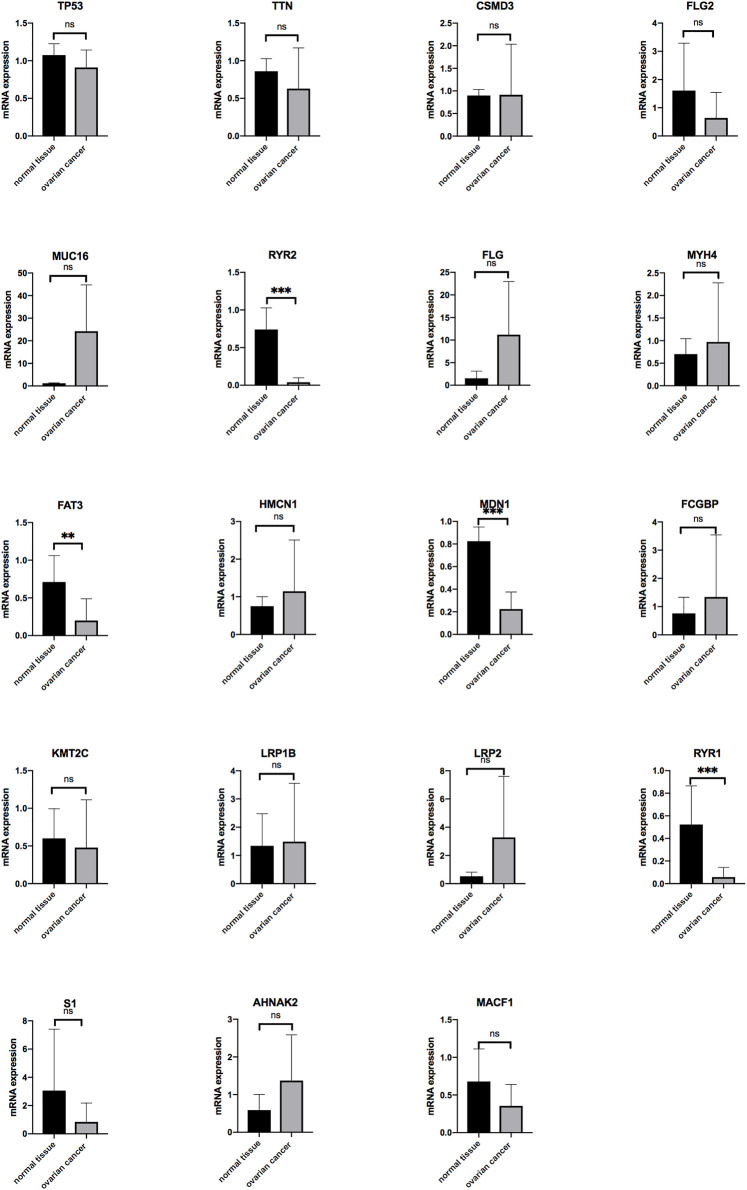
The mRNA expression level of the genes in OV (*n* = 18) and normal ovarian tissues (*n* = 5) (*p < 0.05, **p < 0.01, ***p < 0.001).

To further validate the mutation genes at the mRNA level, qRT-PCR was used in on 18 OV tissues, 5 normal tissues, and 4 OV cells lines. The results indicated that the mRNA expression of RYR2, FAT3, MDN1 and RYR1 was significantly down-regulated in OV compared with normal tissues. The mRNA expression of MUC16, FLG, MYH4, HMCN1, FCGBP, LRP1B, LRP2 and AHNAK2 level tended to increase in the OV compared with the normal tissues but without statistical significance. The mRNA expression of TP53, TTN, FLG2, KMT2C, S1 and MACF1 level tended to decrease in the OV compared with the normal tissues but without statistical significance. The mRNA expression of USH2A was not detected in OV tissues. The expressions of genes in OV and normal tissues are shown in Figure 7.

**FIGURE 8:**
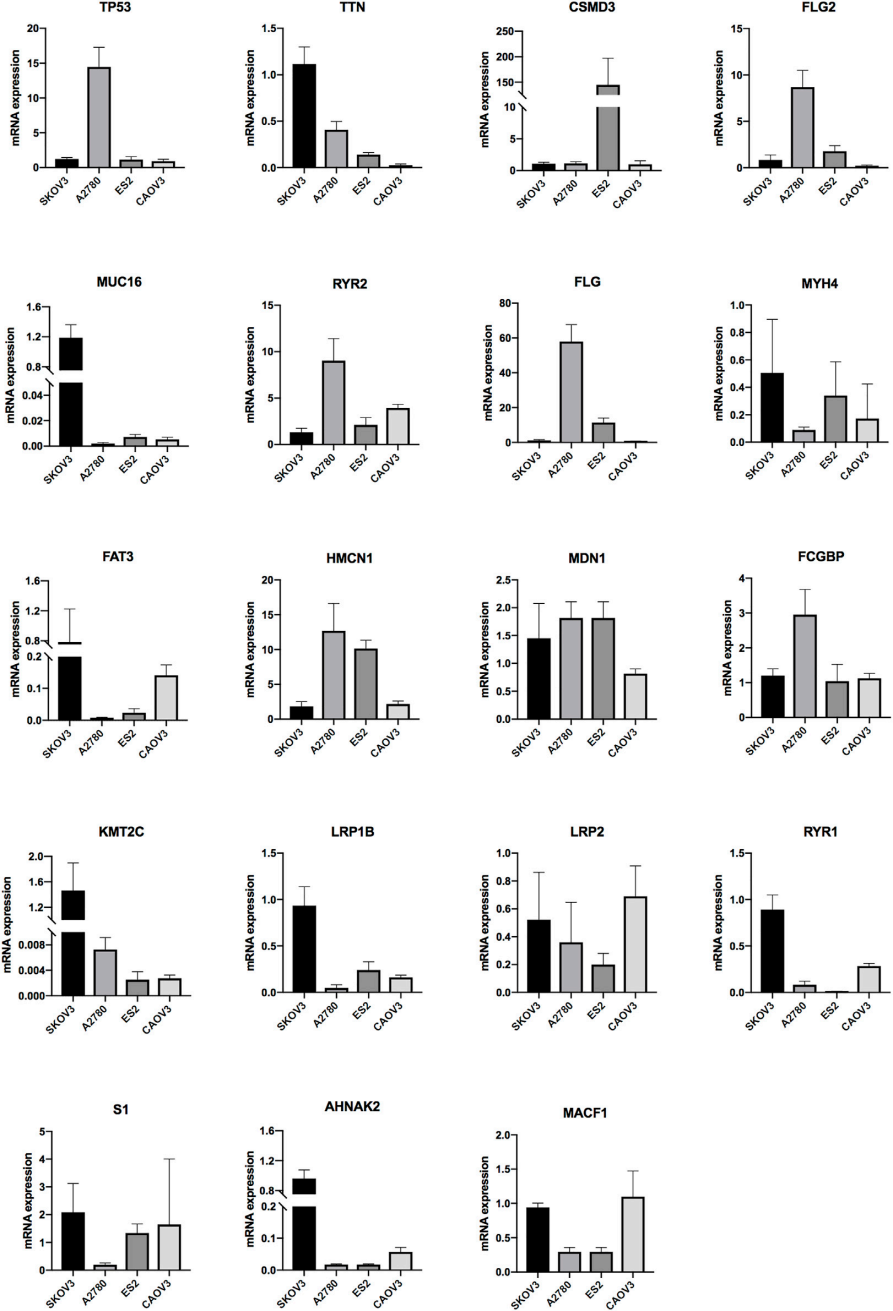
The mRNA expression level of the genes in OV ovarian cells (SKOV3, A2780, ES2 and CAOV3) (*p < 0.05, **p < 0.01, ***p < 0.001).

The expressions of the mutation genes were validated by qRT-PCR in four OV cells (SKOV3, A2780, ES2 and CAOV3). The results showed that TTN, MUC16, MYH4, FAT3, KMT2C, LRP1B, RYR1, S1 and AHNAK2 were highly expressed in SKOV3 cell lines. TP53, FLG2, RYR2, FLG, HMCN1 and FCGBP were highly expressed in A2780 cell lines. CSMD3 and MDN1 were highly expressed in ES2 cell lines. LRP2 and MACF1 were highly expressed in CAOV3 cell lines. USH2A mRNA expression was not detected in all OV cells (Figure 8).

### 3.6 Validation of the Immune Microenvironment Analysis of TCGA OV Samples in ICGC

We used a total of 81 samples from ICGC OV-AU data as a validation set to confirm our results of the TCGA OV tumor microenvironment analyses.

**FIGURE 9:**
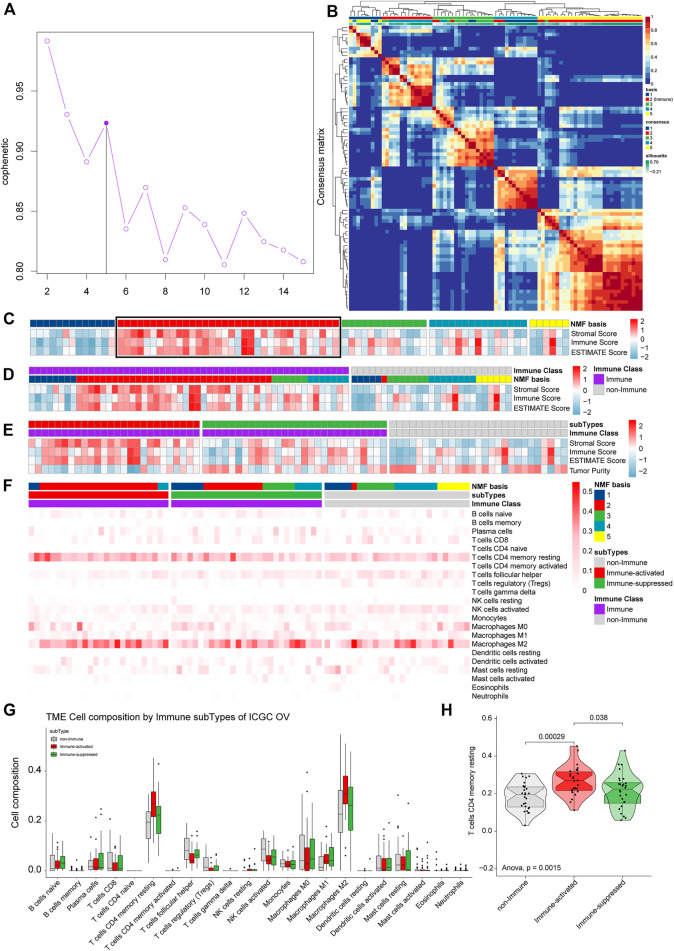
Validation of the immune microenvironment analysis of TCGA OV samples using ICGC. **(A)** The cophenetic values varied with K = 2 to 15 in NMF analysis. **(B)** The consensus map of NMF analysis results from the dimensional reduction of original matrices and consensus analysis among modules. **(C)** The heat map of the NMF clustering results and ESTIMATE analysis results of OV. **(D)** All the samples were classified as Immune Class and non-Immune Class using NMFConsensus analysis. **(E)** ICGC OV-AU dataset was divided into the immune-suppressed subtype, the immune-activated subtype, and the non-Immune Class using NTP analysis. **(F)** The proportions of specific 22 immune cells in each group from NMF analysis. **(G)** The proportions of specific 22 immune cells in the non-Immune Class, the Immune-suppressed subtype, and the Immune-activated subtype. **(H)** The differential proportions of memory CD4 resting T cells among the non-Immune Class, the Immune-suppressed subtype, and the Immune-activated subtype.

The total ICGC OV-AU expression profiles were clustered into five groups by NMF. Then, we estimated the optimal K and found that the cophenetic value changed the most when K varied from 5 to 6 (Figure 9A). Therefore, the optimal rank of 5 was chosen for NMF decomposition (Figure 9B) with 13 samples in factor-1, 34 samples in factor-2, 13 samples in factor-3, 15 samples in factor-4, and six samples in factor-5, where factor-2 was considered to be the Immune factor.

ESTIMATE analysis, including all samples, was conducted and integrated with the NMF results to estimate Immune Factor (Figure 9C). We classified the validation set into an Immune Class with 54 samples and a non-Immune Class with 27 samples. Consistent with the TCGA OV analysis results, the Immune Class contained almost all Immune Factors and higher Immune Scores than the non-Immune Class (Figure 9D). The Immune Class was further grouped into the Immune-activate subtype with 26 samples and Immune-suppressed subtype with 28 samples by NTP analysis (Figure 9E).

To identify the immune microenvironment of OV samples in ICGC, we calculated the proportions of 22 immune cells in OV samples from ICGC. The most common immune cell type was M2 macrophages, followed by memory CD4 resting T cells (Figure 9G). We calculated the proportions of the specific 22 immune cells in each subgroup from (Figure 9F). The Immune-activated subtype had higher proportions of memory CD4 resting T cells than the Immune-suppressed subtype (*p* = 0.038) and the non-Immune Class (*p* = 0.00029) (Figure 9H), which is consistent with the results from TCGA OV analysis.

### 3.7 Validation of the Immune Microenvironment Analysis of TCGA OV Samples in ArrayExpress

**FIGURE 10:**
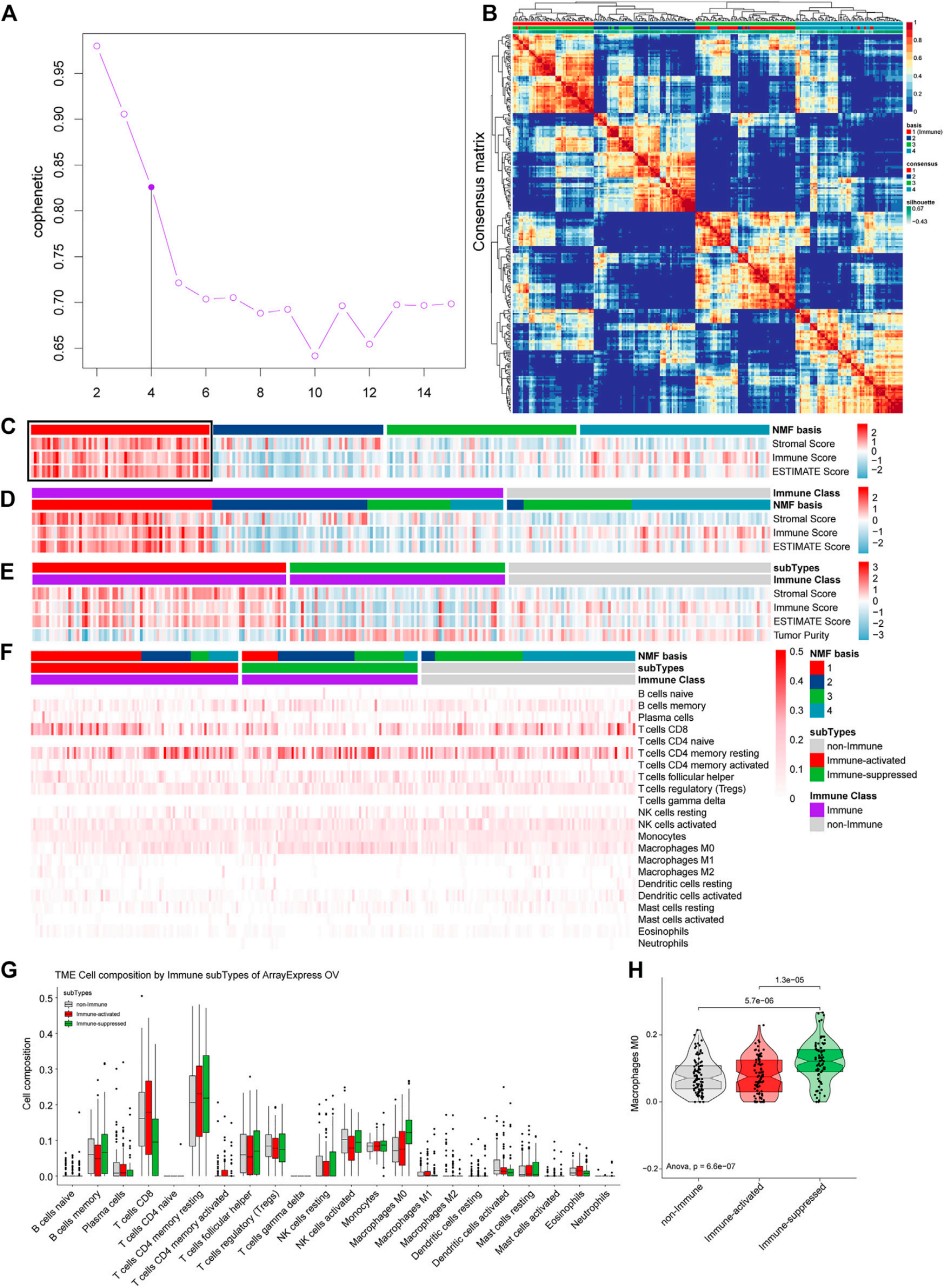
Validation the immune microenvironment analysis of TCGA OV samples with the gene symbol expression profiles from ArrayExpress. **(A)** The cophenetic values varied with K = 2 to 15 in NMF analysis. **(B)** The consensus map of NMF analysis results from the dimensional reduction of original matrices and consensus analysis among modules. **(C)** The heat map of the NMF clustering results and ESTIMATE analysis results of OV. **(D)** All the samples were classified as Immune Class and non-Immune Class using NMFConsensus analysis. **(E)** ArrayExpress gene symbol expression profiles were divided into the immune-suppressed subtype, the immune-activated subtype, and the non-Immune Class using NTP analysis. **(F)** The proportions of specific 22 immune cells in each group from NMF analysis. **(G)** The proportions of specific 22 immune cells in the non-Immune Class, the Immune-suppressed subtype, and the Immune-activated subtype. **(H)** The differential proportions of memory CD4 resting T cells among the non-Immune Class, the Immune-suppressed subtype, and the Immune-activated subtype.

The ArrayExpress gene symbol expression profiles were further used to validate our TCGA OV tumor microenvironment analysis results. We estimated the optimal K and found that the cophenetic value changed the most when K varied from 4 to 5 by NMF (Figure 10A). Therefore, the optimal rank of 4 was chosen for NMF decomposition, and 65 samples in factor-1, 62 samples in factor-2, 69 samples in factor-3, and 69 samples in factor-4 were identified by NMF analysis of 264 samples (Figure 10B), where factor-1 was considered to be the Immune factor. ESTIMATE analysis was conducted integrated with the NMF results to estimate Immune Factor (Figure 10C). We grouped 170 samples in the Immune Class and 95 samples in the non-Immune Class by NMFconsensus analysis. The Immune Class contained almost all Immune Factors and higher Immune Scores of the Immune Class than those of non-Immune Class (Figure 10D), which agrees with the results from TCGA OV analysis.

The 170 samples in the Immune Class were further grouped into 92 samples as the Immune-activate subtype and 78 samples as the Immune-suppressed subtype (Figure 10E).

We calculated the proportions of 22 immune cells in OV samples for the ArrayExpress gene symbol expression profiles to identify the immune microenvironments. The most common immune cell type was memory CD4 resting T cells, followed by CD8 T cells (Figure 10G). We calculated the proportions of the specific 22 immune cells in each group from NMF analysis (Figure 10F). The Immune-suppressed subtype had higher proportions of M0 macrophages than the Immune-activated subtype (*p* = 0.000013) and the non-Immune Class (*p* = 0.0000057) (Figure 10H), consistent with the results from TCGA OV analysis.

## 4 Discussion

The current study aimed to comprehensively analyze the heterogeneous OV immune microenvironment subtypes underlying total immune genes. For this purpose, we analyzed 361 samples based on the TCGA OV dataset. We validated them using two external datasets, 81 samples from the ICGC OV dataset and 264 samples from the ArrayExpress OV dataset. We found the optimal rank as 4 in NMF analysis with the training set containing 361 samples in TCGA OV data. We obtained four factors: factor-1 had 74 samples, factor-2 had 80 samples, factor-3 had 69 samples, and factor-4 had 138 samples. Factor-2 was considered an Immune factor. We further grouped samples into two immune-related classes, among which the Immune Class had 244 samples and the non-Immune Class had 117 samples. The Immune Class was further divided into two subtypes by NTP analysis, of which the Immune-activate subtype had 121 samples, and the Immune-suppressed subtype had 123 samples. We identified 740 DEGs by comparing the two classes. The functional enrichment analysis of the 740 DEGs showed that immune-related, cancer-related, inflammation-related biological functions and pathways were enriched. There were significant differences in the amplification and deletion of genes between the Immune Class and non-Immune Class. The immune-activated subtype had increased proportions of memory CD4 resting T cells, CD8 T cells than the non-Immune Class, M1 macrophages, and M2 macrophages than the Immune-suppressed subtype and the non-Immune Class.

The Metascape database found the top 150 Immune Genes enriched in many immune-related GO items; the most significant enrichment item was the negative regulation of the humoral immune response. This result was consistent with cancer mediating immunosuppression and also suggested that our classification was accurate. Several studies demonstrated the biological function of humoral immunity in OV. A recent study found that CD19^+^ B cells within the total tumor were associated with better overall survival in high-grade serous OV than without CD19^+^ B cells. Tumor B-cell-derived IgA in the OV microenvironment induced OV cell death by redirecting myeloid cells against extracellular oncogenic drivers (Biswas et al., 2021). Most B cells presented in lymphoid structures in the stroma of high-grade serous OV metastases. They could improve the cytotoxic immune response to tumor cells through the secretion of cytokines and chemokines, which helped recruit and support antigen-presenting cells. Besides, immunoglobulins IgGs produced by B cells could target tumor antigens and form immune complexes that were helpful to activate antigen-presenting cells in the TME (Montfort et al., 2017). Thus, these results suggested that immunobiological pathways might be responsible for OV progression, and immunotherapy is a promising treatment strategy.

There were also many significant cancer-related GO enrichment elements in the top 150 Immune Genes, including integrin-mediated cell adhesion, histone H3-K9 modification, double-strand break repair by homologous recombination. Integrin plays an extensive role in cancer progression (Gu et al., 2017; Hou et al., 2020). In OV, it was reported that integrin is associated with proliferation, migration, invasion, chemoresistance, stemness, TIME, etc (Wu et al., 2020; Yan et al., 2021; Yin et al., 2021). The integrin-mediated adhesion was considered part of the metastasis. The methylation of histone H3K9 has been implicated in the development of various cancers due to its involvement in the transcriptional inactivation of chromatin and the induction of expression of cancer suppressor genes (Casciello et al., 2015). Homologous recombination is a key pathway involved in repairing DNA double-strand breaks. Konstantinopoulos et al. (2015) reported that around 50% of epithelial ovarian cancers are deficient in DNA repair via homologous recombination. New studies revealed that deficient DNA repair *via* homologous recombination is associated with immunotherapy response (Mouw et al., 2017). In the Keynote-162 (NCT02657889) phase I/II trial, 62 platinum-resistant recurrent OV patients were treated with pembrolizumab plus the inhibitor niraparib. The study reached an ORR (primary endpoint of the study) of 25% and a disease-control rate (DCR) of 68% (Konstantinopoulos et al., 2019). Therefore, the immune genes in OV might play various biological functions rather than immunity, indicating that cross-talks existed in immunobiological and other biological pathways.

Through the GO enrichment analysis of DEGs by comparing the Immune Class with the non-Immune Class, we found that many biological functions were highly enriched in immune-related processes, including immune response, immune response regulation, cell-cell signaling, leukocyte migration, signal transduction, cellular defense response, and adaptive immune response. Besides, KEGG enrichment analysis showed that the DEGs were significantly enriched in the hematopoietic cell lineage, the cytokine-cytokine receptor interaction, the primary immunodeficiency, and allograft rejection. These results indicated that DEGs played a potential role in regulating the TIME and greatly supported that the TIME was involved in OV malignant progression, consistent with previous studies (Westergaard et al., 2020; Shen et al., 2021). Moreover, it provided supporting evidence that both the Immune Class and the non-Immune Class existed in the OV immune microenvironment and differed significantly in their biological functions.

Inflammation is a hallmark of cancer, and the inflammatory state of premalignancy promotes tumor progression by various immune cells (Hanahan and Weinberg, 2011). In the present study, we performed GO analysis and identified inflammatory response as the most significant GO enrichment term of DEGs. KEGG pathway analysis showed that DEGs were significantly enriched in *Staphylococcus aureus* infection, phagosomes, asthma, viral myocarditis, and malaria. Numerous studies have shown that systemic inflammatory response markers, such as the neutrophil to lymphocyte ratio, and the platelet to lymphocyte ratio, could provide useful prognostic information among patients with OV (Zhang et al., 2017; Kwon et al., 2018; Yoshida et al., 2019). Interestingly, we also found that extracellular matrix organization and cell adhesion were enriched in GO analysis, and ECM-receptor interaction and cell adhesion molecules were enriched in KEGG analysis. These terms reflected the cross-talks that existed between inflammation and angiogenesis (Szewczyk et al., 2019) and between inflammation and metastasis (DiGiacomo and Gilkes, 2018). Angiogenesis is a common and critical biomarker of the inflammation-to-cancer transition in cancers. The infiltrated immune cells and their secreted cytokines were partly responsible for the inflammation-to-cancer transition. Furthermore, inflammation-to-cancer cytokines and angiogenesis genes might serve as predictors of survival and immune therapy response (Chen et al., 2019). Accordingly, it is suggested that the combination therapies of immunotherapy with anti-inflammatory therapy and anti-angiogenesis therapy might be possible and efficient.

In GSEA analysis, we discovered the NOD-like receptor signaling pathway, the Toll-like receptor signaling pathway, and many immune cells and immune response associated pathways significantly enriched in the Immune Class. A recent study demonstrates that activation of Toll-like receptor 8 reversed the immunosuppression function of regulatory T cells (Tregs) among TME by regulating glucose metabolism of Tregs in OV (Xu et al., 2021). Toll-like receptor 4 signaling pathway promoted OV cell proliferation and metastasis by activating osteopontin (Xu et al., 2017). Thus, it provided new insights for OV treatment and predictive prognosis.

The CNV-based risk score is an independent and discriminatory biomarker for the survival of OV patients (Graf et al., 2021). We also explored the CNV differences between the Immune Class and non-Immune Class. The amplification of 8q24.21 was detected in both Immune-activated and Immune-suppressed subtypes. The amplification of MYC, one of the genes located on 8q24.21, increased the sensitivity of OV cells to PARP inhibitors (Papp et al., 2018). TP53 was the most common mutated gene among all OV samples, and there was no difference among the three subtypes, likely due to the mutations in the TP53 gene in a high proportion (70%) of OV. This observation was consistent with a previous study (Cancer Genome Atlas Research, 2011). These results suggested the CNVs of the Immune Class might be closely associated with immunobiological pathways, further providing new targets for immunotherapy.

Notably, it has been previously reported that high levels of various immune cells are infiltrated into the TME of OV (Wang et al., 2018). Our independent analysis consistently discovered the immune cells expressed in tumors, especially memory CD4 resting T cells, macrophages M0, and macrophages M2. Memory CD4 resting T cells expression was associated with the prognosis in patients with OV (An and Yang, 2020). Lampert *et al.* (Lampert et al., 2020) found that memory CD4 resting T cells increased after treatment with a checkpoint 1 inhibitor of the cell cycle, perhaps due to the adaptive immune response activation. M0 macrophages conferred favorable OS, whereas the M2 macrophages were correlated with a poor OS in OV (Liu et al., 2020). M2 Macrophages inhibited apoptosis and increased the proliferation, invasion, and migration of OV cells (Yi et al., 2020). Recently, one study revealed that M2 macrophages controlled the vascular barrier by regulating the VCAM1/RAC1/ROS/p-PYK2/p-VE-cad axis. According to our results, the immune cells in the TIME played critical roles in OV progression, indicating the possibility of immunotherapy for OV.

Additionally, we also validated the aforementioned immune microenvironment analysis of TCGA OV samples in ICGC and ArrayExpress. All the results were as expected, suggesting that our results provided an accurate and repeatable classification of OV samples, as well as the potential targets for immunotherapy and biomarkers to predict outcomes.

OV is an aggressive malignancy that is still one of the most lethal gynecological cancers worldwide (Sung et al., 2021). The advanced stage was diagnosed in seventy-five percent of OV patients (Ferlay et al., 2015). The etiology of OV is unclear, and the major challenge in the clinical management of OV is the lack of effective treatment options. OV is currently stratified into different subtypes based on clinical and pathological characteristics. In contrast, the OS of different subtypes varies, indicating that biological heterogeneity still exists within each subtype. Therefore, it is necessary to investigate the underlying molecular subtypes according to specific gene patterns to evaluate and improve individualized medical decisions for OV patients. Taking advantage of the development of bioinformatics in recent years, especially the high-throughput sequencing technology, it is possible to systematically research the underlying mechanism of OV at the genome level.

OV is considered to be immunogenic, however, several clinical studies did not show a promising benefit for immunotherapy in OV recently (Moore et al., 2021; Pujade-Lauraine et al., 2021). Despite some OV patients show response to immunotherapy, there remains a subset of patients with PD-L1 expression who do not respond. Several markers have been found to be related to the efficacy of immunotherapy, such as TMB, PD-1, PD-L1, homologous repair deficient and proficient, TME and TILs (Conway et al., 2018; Keenan et al., 2019; Pellegrino et al., 2020; Plesca et al., 2020; Paijens et al., 2021). These factors were also been found in the present study. There are still lots of problems about the complex network of the immunogenicity of the cancer and the TIME needs to be further investigated, for example, how the immune system accesses the tumor, how the immune cells perform the killing functions, which is the ideal markers for response to immunotherapy. The present study improved understanding of immune interactions of OV and TIME.

We also performed external validation with the OV tissues and cells. The qRT-PCR results showed that the mRNA expression levels of FAT3, a putative tumor suppressor gene which codes for an atypical cadherin, were down-regulated in OV tissues. A study showed that FAT3 was enriched with strong mutations in metastases lesions in OV patients (Ojasalu et al., 2020). A recent study showed that the lung cancer patients with co-mutation of FAT3 and LRP1B had significantly prolonged immunotherapy PFS, which indicated that co-mutation of FAT3 and LRP1B is a promising biomarker to predict the efficacy of immunotherapy (Zhu et al., 2021).

However, our study may have some limitations. First, although three independent datasets were involved in the present study, more samples are still needed for comprehensive analysis to combat bias. Secondly, more clinical data should be included to analyze the immune signature and clinical characteristics comprehensively. Thirdly, more experimental evidence is needed to explain the molecular mechanisms and biological significance of our immunogenomic analysis.

In conclusion, we clustered the OV patients into three subtypes. The three subtypes of OV patients showed distinct gene signatures and TIME status, and the classification was efficient and repeatable. Moreover, novel functional genes and pathways that might contribute to the TME of OV were identified. Thus, the current study provided potential clues for further research on the molecular mechanisms and immunotherapy strategies of OV.

## Data Availability

Publicly available datasets were analyzed in this study. This data can be found here: TCGA (https://www.cancer.gov/about-nci/organization/ccg/research/structural-genomics/tcga), ICGC (https://dcc.icgc.org/), and ArrayExpress (https://www.ebi.ac.uk/arrayexpress/). The R code for this article is available at https://github.com/wangjianliu1203/Immune-signatures-for-ovarian-cancer.git.

## Glossary

ANOVA: analysis of Variance
BP: biological process
CNVs: copy number variations
CTLA-4: cytotoxic T-lymphocyte antigen-4
DAVID: Database for Annotation, Visualization and Integrated Discovery
DCR: disease-control rate
DEGs: differentially expressed genes
DMEM: Dulbecco’s modified Eagle medium
ESTIMATE: Estimation of STromal and Immune cells in MAlignant Tumor tissues using Expression data
FBS: fetal bovine serum
FC: fold-change
GO: gene ontology
GSEA: gene set enrichment analysis
HGNC: Human Genome Organization’s Gene Nomenclature Committee
HR: hazard ratio
ICGC: International Cancer Genome Consortium
KEGG: Kyoto Encyclopedia of Genes and Genomes
LM22: 22 immune cell subtypes
MAF: mutation annotation format
NMF: Non-negative Matrix Factorization
NTP: nearest template prediction
ORR: objective response rate
OS: overall survival
OV: ovarian cancer
OV-AU: Australian Ovarian Cancer Study cohort
PCA: principal component analysis
PD-1: programmed cell death protein 1
PFS: progression-free survival
qRT-PCR: Quantitative Real-Time PCR
RMA: Robust Multi-array Average
SD: standard deviation
SNPs: single nucleotide polymorphisms
TCGA: the Cancer Genome Atlas Program
TILs: tumor-infiltrating lymphocytes
TIME: tumor immune microenvironment
TMB: tumor mutation burden
TME: tumor microenvironment
