# Supplementary material for: Identification of the Immune Signatures for Ovarian Cancer Based on the Tumor Immune Microenvironment Genes
Source: Front Cell Dev Biol. 2022 Mar 17;10:772701. doi: 10.3389/fcell.2022.772701 (PMC8974491; doi:10.3389/fcell.2022.772701)
Supplement: Supplementary file 1 [file Table1.DOCX]

Table 1 Primer sequences

| **ID** | **Primer** |  | **Sequence (5' to 3')** |
| --- | --- | --- | --- |
| 1 | TP53 | FORWARD | GCCCATCCTCACCATCATCACAC |
|  | TP53 | REVERSE | GCACAAACACGCACCTCAAAGC |
| 2 | TTN | FORWARD | GGTTCCAAGTACAGGCTATCCAAGG |
|  | TTN | REVERSE | ACAAGTTCGGCATAGGCAGACAAG |
| 3 | CSMD3 | FORWARD | GAGCATCACCTGTCAACGGATAGC |
|  | CSMD3 | REVERSE | GGTCCTTGAAGATTAGAGCCACACG |
| 4 | FLG2 | FORWARD | GGGCACTCAGGGTTCTCACAAAG |
|  | FLG2 | REVERSE | TGTCCACTGGTATCTCCTGTCTGTC |
| 5 | MUC16 | FORWARD | CATTACCAGCAAGTAGCCACTCCTC |
|  | MUC16 | REVERSE | CGTCCAACACCTCAGTAGTCTTCAC |
| 6 | RYR2 | FORWARD | CTGGAACTGGACACGCCTTCTATTG |
|  | RYR2 | REVERSE | CCTCTGCTGCCACCATCAAACTC |
| 7 | FLG | FORWARD | CCATCACAGCCACACCACATCC |
|  | FLG | REVERSE | GTGCCGTCTCCTGATTGTTCCTC |
| 8 | USH2A | FORWARD | TAAAGTCATTCCCATCCTGGTG |
|  | USH2A | REVERSE | CCAGAGCTGCATATTTTCCTTC |
| 9 | MYH4 | FORWARD | CTGATGACCAACTTGAGGAGCACTC |
|  | MYH4 | REVERSE | GCCTTCCAGCACACCGTTACAC |
| 10 | FAT3 | FORWARD | TGAGCAGCAGTCTTCGTACAACATC |
|  | FAT3 | REVERSE | CAGAACGGTGATGGTGACAGTAGTG |
| 11 | HMCN1 | FORWARD | TGCTGAATGAGACTGTGTTGGTGAG |
|  | HMCN1 | REVERSE | TGCTGGTGGAACCTGAGAGTAGAC |
| 12 | MDN1 | FORWARD | ACAAGGCAGTGACGCATACGATG |
|  | MDN1 | REVERSE | GGTGTCCTCTATCTCCTCCTCTTCC |
| 13 | FCGBP | FORWARD | TGCCCACCTCCTCATGTCCATC |
|  | FCGBP | REVERSE | AAGTCGTCACTCCAGTTGCCATTG |
| 14 | KMT2C | FORWARD | GTCTCCACAACACCAACAGCAAATG |
|  | KMT2C | REVERSE | CTACCTGCCTTCGCTCATTAGTCTG |
| 15 | LRP1B | FORWARD | CACTGCGACTCTGATGACGACTG |
|  | LRP1B | REVERSE | ATCTGCCACTGGAACATCTGAACTG |
| 16 | LRP2 | FORWARD | TCGGAGACAGGTGATAGCCAGTG |
|  | LRP2 | REVERSE | ACGGTCAGTCCAGTACACAGAGTC |
| 17 | RYR1 | FORWARD | TTCTTCGTCATCGTCATCCTGTTGG |
|  | RYR1 | REVERSE | TCCTTCACTTGCTCTTGTTGGTCTC |
| 18 | SI | FORWARD | ACAGGCAAAGATATTGGCGTCAGAG |
|  | SI | REVERSE | GCTGGCTCTTGACATGGTAGGATG |
| 19 | AHNAK2 | FORWARD | AGGTCTCGGTGGATGTGTCTGC |
|  | AHNAK2 | REVERSE | GAATGCGGAGGTCAGTGGTCTTG |
| 20 | MACF1 | FORWARD | ccaccctcattcagcgggat |
|  | MACF1 | REVERSE | cgtcaggctgtttgcgagtc |
| 21 | GAPDH | FORWARD | AAGGTGAAGGTCGGAGTCAAC |
|  | GAPDH | REVERSE | GGGGTCATTGATGGCAACAATA |
